# Modular structure within groups causes information loss but can improve decision accuracy

**DOI:** 10.1098/rstb.2018.0378

**Published:** 2019-04-22

**Authors:** Albert B. Kao, Iain D. Couzin

**Affiliations:** 1 Santa Fe Institute, Santa Fe, NM 87501, USA; 2 Department of Collective Behaviour, Max Planck Institute for Ornithology, 78464 Konstanz, Germany; 3 Chair of Biodiversity and Collective Behaviour, Department of Biology, University of Konstanz, 78457 Konstanz, Germany; 4 Centre for the Advanced Study of Collective Behaviour, University of Konstanz, 78457 Konstanz, Germany

**Keywords:** collective behaviour, collective decision-making, modular structure, subgrouping, information correlation

## Abstract

Many animal groups exhibit signatures of persistent internal modular structure, whereby individuals consistently interact with certain groupmates more than others. In such groups, information relevant to a collective decision may spread unevenly through the group, but how this impacts the quality of the resulting decision is not well understood. Here, we explicitly model modularity within animal groups and examine how it affects the amount of information represented in collective decisions, as well as the accuracy of those decisions. We find that modular structure necessarily causes a loss of information, effectively silencing the input from a fraction of the group. However, the effect of this information loss on collective accuracy depends on the informational environment in which the decision is made. In simple environments, the information loss is detrimental to collective accuracy. By contrast, in complex environments, modularity tends to improve accuracy. This is because small group sizes typically maximize collective accuracy in such environments, and modular structure allows a large group to behave like a smaller group (in terms of its decision-making). These results suggest that in naturalistic environments containing correlated information, large animal groups may be able to exploit modular structure to improve decision accuracy while retaining other benefits of large group size.

This article is part of the theme issue ‘Liquid brains, solid brains: How distributed cognitive architectures process information’.

## 1. Introduction

From choosing where to invest time searching for food to deciding whether to fight or flee from a predator, an animal’s decisions may impact its probability of survival and reproduction. Animals have therefore evolved mechanisms to exploit available information in order to improve their decisions. Many organisms do not make such decisions in isolation, however. The social environment in which many animals live (and make decisions) can also strongly influence the decision-making abilities of individuals. Indeed, a large and growing body of theoretical and empirical evidence suggests that making decisions collectively can often improve decision accuracy across a wide range of contexts, including detecting the presence of predators [1,2], locating the correct direction in which to migrate [3,4], when climbing environmental gradients [5,6] and discovering energy-efficient travelling routes [7,8]. Similar results have been obtained in several domains of human decision-making, including forecasting future events [9], forming medical diagnoses [10] and estimating numerosities [11–13], suggesting that the mechanisms underlying collective decision-making may be general and find broad applications across contexts and species.

One such collective decision-making mechanism involves the pooling of information across individuals in the group [3,4]. Because each group member necessarily occupies a unique position in space, and therefore experiences somewhat unique stimuli from the environment, the information available to the group as a whole can be much greater than that sensed by any single individual [14,15]. It has therefore been hypothesized that the collective decision generated by such a distributed sensory array has the potential to outperform individual decisions, simply owing to the greater amount of information available to the group [14].

However, the decision-making benefits of information pooling require some diversity of information among the individuals, since there can be no potential for improvement in decision accuracy if all individuals possess identical information. In reality, rather than being completely independent of each other, the information perceived by animals in a group will often be correlated to some degree. This can arise owing to intrinsic spatial correlations in environmental cues (e.g. odour plumes, sounds or visual stimuli) [16,17]. Another mechanism by which opinions can be correlated is if individuals can be influenced by the opinions, or behaviour, of others through social learning [11,18]. Such correlations in information across individuals have been shown to degrade collective accuracy generally [19–23], and recent research has demonstrated that small groups often maximize collective accuracy in such scenarios [16,18]. Increasing group size initially allows the individuals to exploit the benefits of information pooling, but increasing the size of the group further causes correlations (either from the environment or from social influence) to dominate the collective decision-making and consequently degrade accuracy [16].

Models that seek to explain the benefits of collective decision-making, whether in relatively simple environments or scenarios involving correlations, typically assume, either explicitly or implicitly, that individuals in groups make decisions through simple majority rule, or a related rule such as a weighted majority [24] or quorum rule [1,25–28]. Indeed, majority rule can be mapped onto many real animal decision mechanisms [29–32], and even models of collective movement, where individuals are typically assumed to interact only with near neighbours, produce overall decisions that closely match majority rule [17,24,33]. In short, animal groups are often assumed to be well-mixed over the course of a decision, such that the resulting decision is well approximated by simple majority rule.

By contrast, however, rather than being well-mixed, many real animal groups exhibit signatures of persistent modular structure in the group, whereby individuals interact with certain other individuals more than others. Early studies of fish schools in the laboratory and in the field described subsets of the school moving semi-independently [34–37], often forming appendages at the edges of the school [38] or ‘lacunae’ within the school [39–43]. Distinct subgroups within the larger school have been observed in a wide range of fish species, including saithe [39,44], herring [35,45], mackerel [46], capelin [47] and minnows [42], through the observation of correlations in movement across individuals, or the detection of high density regions within a school. In these studies, for schools ranging in size from 12 to 70 fish, subgroups tended to consist of two to five individuals [46]. Similarly, in birds, subgroups have been observed in rock doves [48] and domains of correlated movement have been identified in starlings [49].

More broadly, many animal species can form complex hierarchical structures within a social group, or societies within a larger population [50]. By characterizing the social networks of animal groups (e.g. [51,52]) or using other methods, researchers have identified such complex social structures across diverse taxa, including primates [53,54], bats [55], hyenas [56], equids [52], cetaceans [57], elephants [58] and birds [59]. In such hierarchical structures, a particular animal often forms close alliances with certain individuals (such as kin), but maintains relatively looser affiliations with others, either within the same group, or across distinct groups. A number of proximate mechanisms, including individual recognition and self-sorting (without the need for individual recognition) [51,60,61] have been found to be capable of generating persistent non-random associations between individuals.

Furthermore, many social insects, such as ants [62,63], honeybees [64], bumblebees [65] and wasps [66], interact more frequently with certain individuals in the colony than other individuals. This spatial heterogeneity can strongly affect how information about food availability or environmental conditions spreads across the colony. However, because collective decisions in such colonies also use other mechanisms, such as pheromones [67], waggle dances [68] and recruitment [69]—often physically embodied outside of the nest site—in this study we focus on modelling other animal groups, such as fish schools and bird flocks, rather than social insects in particular.

In the context of this special issue, animal groups exhibiting some modular structure inhabit an intermediate space between ‘liquid brains’ (in which the links connecting components are highly dynamic) and ‘solid brains’ (in which the links are static), exhibiting some features of both. Here, we systematically examine the effect of such intermediate structure on the quality of collective decision-making by group-living animals. To facilitate this analysis, we define a new measure of the information contained within a collective decision, the *effective group size* (in analogy to effective population size in population genetics, and effective reproduction number in disease dynamics), which allows for the quantification of the effect of modular structure on the quality of a collective decision. We then analyse the effect of modularity within simple environments, as well as more naturalistic scenarios (environments with multiple cues that vary in correlation [16]) to understand how such internal structure may be strategically exploited by animals in a group to improve collective decision accuracy.

## 2. Defining the effective group size

**Figure 1.**
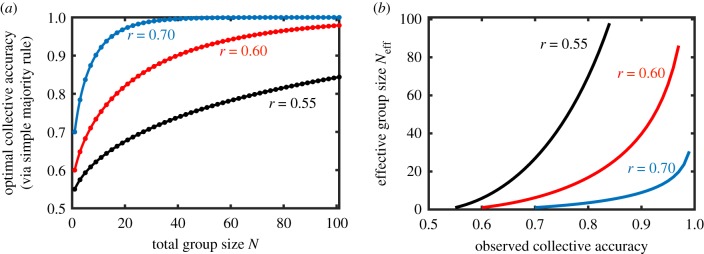
Defining the effective group size. (*a*) A group of individuals, each with a probability *r* of selecting the correct option (out of two available options), can maximize its collective accuracy by combining the individual opinions through simple majority rule. Shown is the collective accuracy resulting from simple majority rule as a function of the total group size *N*. (*b*) By inverting the relationship in (*a*), we define a new measure, the *effective group size* *N*_eff_, to describe the information loss incurred owing to modular structure within the group. The effective group size is the size of the group that, making a decision through simple majority rule, achieves the same observed accuracy as a (larger) group containing some modular structure.

The classic Condorcet jury theorem [70] demonstrated that, given a choice between two options (where one option is inherently superior to the other but is known imperfectly to a group of individuals) in which each individual selects the better option with the same probability *r* > 0.5, then the optimal method to combine the individual opinions is to select the more popular option (i.e. simple majority rule) [71]. Doing so allows for a monotonic increase in collective decision accuracy as group size increases, saturating at perfect accuracy as group size grows very large (figure 1*a*).

Although each individual contributes equally valuable information to the group (i.e. they all have the same individual accuracy *r*), the marginal improvement in collective accuracy owing to the addition of another individual decreases with group size. This nonlinear relationship between group size and collective accuracy makes collective accuracy a suboptimal measure to quantify the effect of modular structure on decision accuracy. To counter this, we define a new measure of information content, the effective group size, by inverting the relationship illustrated in figure 1*a*. Thus, for a particular individual accuracy *r*, we can map any observed collective accuracy, arising from any decision-making process or any internally structured group, to an effective group size *N*_eff_, which is the group size that would achieve the same collective accuracy if making a decision by simple majority rule (figure 1*b*). Given that simple majority rule reflects the optimal use of the available information contained within the individuals in the group in this context, the effective group size will generally be smaller than the actual group size. The ratio between the effective group size *N*_eff_ and the actual group size *N* allows us to quantify how much information is lost owing to the introduction of internal structure, or other details of the collective decision-making process.

## 3. Modelling modular structure within groups

**Figure 2.**
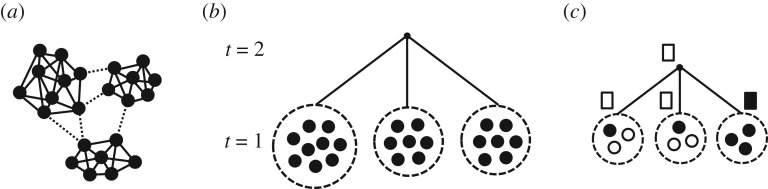
Modelling modular structure in groups. (*a*) Many group-living animals associate closely (solid lines) with certain individuals, and only interact weakly (dotted lines) with other individuals, which can create persistent subgroups within the larger group. (*b*) To model such modular structure in animal groups, we assign each individual to a unique subgroup (dotted circles). The individuals within each subgroup form a consensus opinion through simple majority rule (shown in tier *t* = 1). Following this, the decisions of the subgroups are then combined into an overall consensus decision, also through simple majority rule (tier *t* = 2). An arbitrary number of subgroups and tiers can be modelled in this framework. (*c*) An example demonstrating how a minority can determine the collective decision when modularity is present. While a majority (five out of 11) individuals voted for one option (indicated in black), this particular assortment of individuals into subgroups resulted in a consensus decision for the other option (indicated in white).

Many animals living in social groups associate strongly with a subset of the group, and more weakly with other group members [50,60,61]. As a result, relatively distinct, semi-independent subgroups can often be detected within the group [46]. In such groups, information may be shared among members of a particular subgroup at a relatively fast time-scale, while information is transferred, and a consensus formed, between subgroups at a slower time-scale (figure 2*a*).

To formalize this structure, we consider each individual to belong to a particular, mutually exclusive subgroup (figure 2*b*). A consensus is first formed within each subgroup through simple majority rule, and then the decisions of the subgroups are combined to form an overall collective decision, also through simple majority rule (see §7 for details). To increase the generality of our results, we additionally tested two modifications to our model. One model does not assume that individuals have identical accuracies but rather have accuracies normally distributed around some value, while the other model assumes that the decisions of the subgroups are combined not through simple majority rule but instead are weighted by the size of each subgroup (see §7 for details). In addition, the structure may be generalized from the two-tier system that we examine here, to many tiers, in which subgroups are further divided into smaller units.

Previous theoretical work has shown that, in general, such modular structure causes a loss of information, whereby decisions made in these systems tend to be less accurate compared to decisions made when all group members vote directly through majority rule, without any such internal structure [72–74]. This is because modularity allows the minority preference to be selected occasionally by the group (figure 2*c*). Because the Condorcet jury theorem states that the majority is more likely to be correct than the minority (provided that individual accuracy *r* > 0.5), these instances of ‘majority deficiency’ [74] tend to degrade collective accuracy. In general, because only the majority opinion propagates beyond a subgroup, the minority opinion in each subgroup is silenced at higher levels of the decision-making process. The effective group size is therefore a natural measure of the loss of information caused by the silencing of a certain fraction of the group.

## 4. Modular structure causes information loss

The space of possible modular structures that can be generated spans four dimensions: the total number of individuals in the group *N*; the evenness in the sizes of the subgroups *S* (measured here as the Shannon diversity of the occupancy of the subgroups, normalized by the smallest and largest possible values that the Shannon diversity can take); the accuracy of each individual *r*; and the number of subgroups *M*. Here we assume only two tiers in the hierarchy (i.e. the group is composed of subgroups, with no further division of the subgroups), both for simplicity and because they adequately describe many simpler animal social structures (such as those observed in fish schools [46] and bird flocks [48]).

For each combination of parameter values, we computed the ratio of the effective group size to the actual group size to measure how much information is lost owing to that particular modular structure. For this analysis, we assume, initially, a scenario identical to that of the Condorcet jury theorem, where each individual independently selects the correct of two options with a probability *r*.

**Figure 3.**
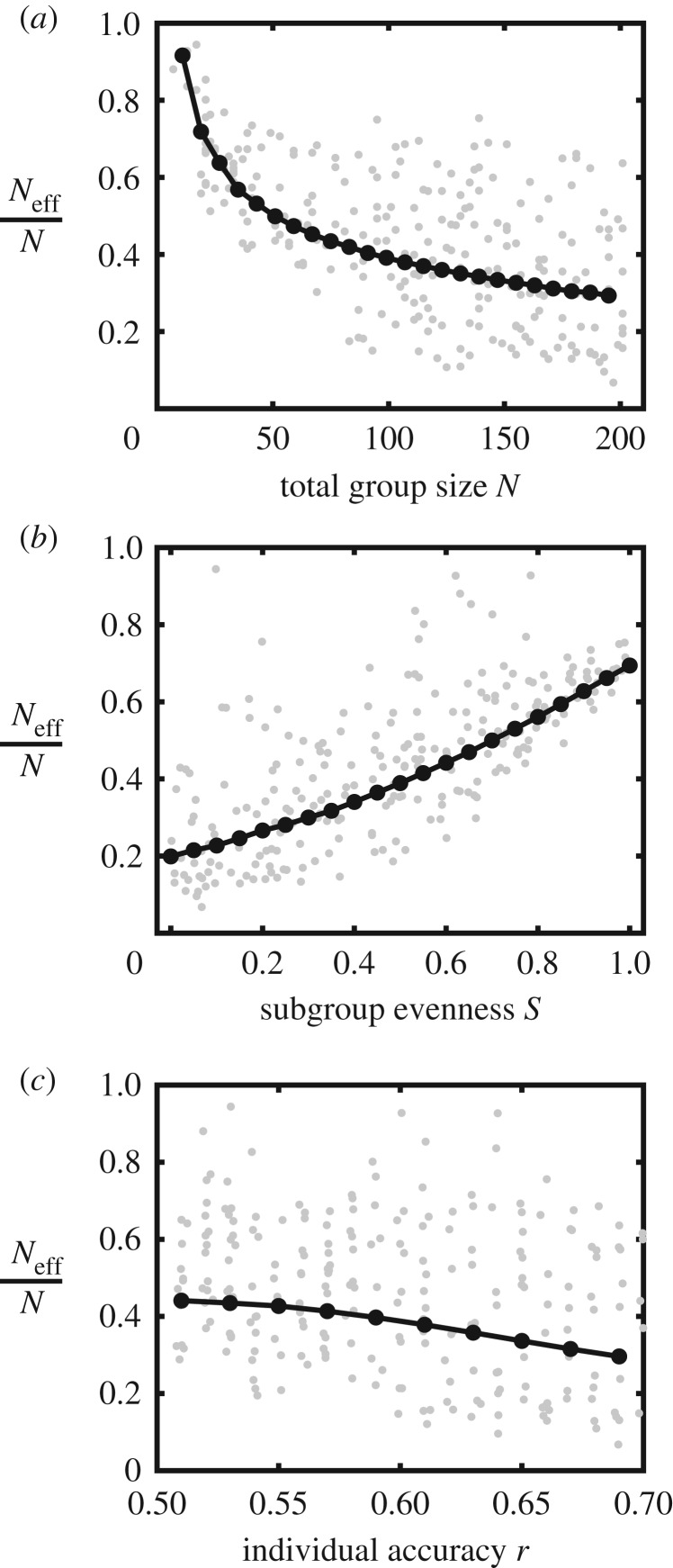
The effects of group size, subgroup evenness and individual accuracy on the effective group size. The ratio of the effective group size to the total group size was computed for random sets of parameter values spanning a region where group sizes *N* ranged from 5 to 201, subgroup evenness *S* ranged from 0 to 1, individual accuracy *r* from 0.51 to 0.70 and number of subgroups *M* from 3 to 15. Shown in grey are the individual data points for each set of parameter values. Shown in black are the effective group size ratios, as a function of (*a*) total group size, (*b*) subgroup evenness and (*c*) individual accuracy, respectively, while holding all other variables at their mean value. Subgroup evenness *S* was calculated relative to the maximum and minimum Shannon diversity possible, where *S* = 0 indicates a maximally uneven distribution (all but one subgroups contain one individual, and one subgroup contains the rest of the individuals) and *S* = 1 indicates a maximally even distribution (subgroups contain equal, or nearly equal, numbers of individuals); see §7 for details of the model, and electronic supplementary material for the Matlab code used to generate all results figures.

We find that the relative effective group size ratio decreases as the total group size increases (figure 3*a*). This is because the collective accuracy tends to saturate and approach 1 at large group sizes, so a small decrease in collective accuracy results in a large drop in the effective group size. The effective group size ratio increases as the subgroups become more equally sized (figure 3*b*), agreeing with earlier work showing that equally sized subgroups tend to outperform asymmetric groups [19,72,75]. We find a mild decrease in the effective group size ratio as the accuracy of the individuals increases (figure 3*c*), for a similar reason to that for total group size.

The results are very similar when individuals have a distribution of accuracies, rather than identical accuracies (electronic supplementary material, figure S1). However, when subgroup decisions are weighted by their size, we find that the effective group sizes tend to be greater overall compared with consensus decisions formed by weighting subgroup decisions equally (electronic supplementary material, figure S2). When subgroup decisions are weighted by their size, the effective group size ratio decreases much less sharply as the total group size increases (electronic supplementary material, figure S2*a*) and is nearly constant as individual accuracy changes (electronic supplementary material, figure S2*c*). However, rather than a monotonic increase in the effective group size ratio as the subgroups become more evenly sized, we find that the effective group size ratio is relatively high when subgroup evenness is very low or very high, and at its minimum when the evenness is moderate (electronic supplementary material, figure S2*b*). This is because highly uneven subgroup sizes (e.g. when there is one very large group and several very small groups) behave similarly to a group with no modular structure since the large subgroup is much more strongly weighted than the small subgroups.

**Figure 4.**
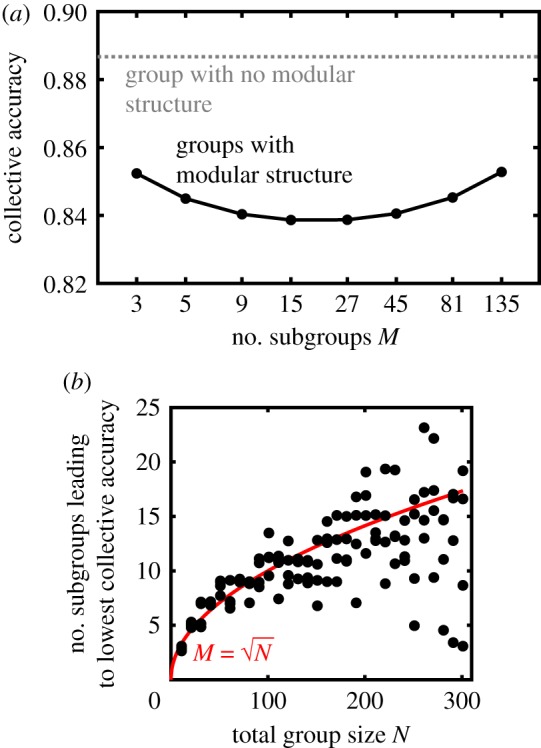
The effect of the number of subgroups on collective accuracy. (*a*) Collective accuracy does not increase monotonically as the number of subgroups increases; instead, an intermediate number of subgroups containing an intermediate number of individuals leads to the lowest collective accuracy. Shown is an example for a group of total size *N* = 405, which can be split into a range of evenly sized subgroups (black), with individual accuracy *r* = 0.53. For comparison is the collective accuracy that would be achieved for a group of the same size, with no modular structure (grey dotted line). The number of subgroups *M* is displayed on a logarithmic axis for clarity. (*b*) The number of subgroups leading to the lowest collective accuracy as a function of total group size *N*, across individual accuracies *r* ranging from 0.55 to 0.70, with subgroup evenness *S* fixed at 1. Overlaid in red is the line $M=\sqrt{N}$. The scatter at large *N* is due to the very high collective accuracy for large groups, especially when *r* is also large, making it computationally difficult to resolve the number of subgroups leading to the lowest accuracy. (Online version in colour.)

Previous work noted that given two numbers, *n*_1_ and *n*_2_, where *n*_1_ > *n*_2_, having *n*_1_ subgroups consisting of *n*_2_ individuals per subgroup leads to higher collective accuracy compared to *n*_2_ subgroups consisting of *n*_1_ individuals per subgroup for the model where individuals have identical accuracy and subgroup decisions are combined through simple majority rule [22,72,76]. This led researchers to conjecture that generally it is better to have a large number of small subgroups than a small number of large subgroups [22,72]. While we find agreement that, for a given pair *n*_1_ and *n*_2_, higher collective accuracy is indeed achieved with a large number of small subgroups than vice-versa, this does not imply that collective accuracy increases monotonically as the number of subgroups increases (figure 4*a*). By contrast, in general we observe that a moderate number of subgroups minimizes collective accuracy. However, the change in collective accuracy loss from adjusting the number of subgroups tends to be relatively small, compared to the loss resulting from introducing any modular structure into the group (figure 4*a*). Nonetheless, across a wide range of group sizes and parameter values (but assuming that subgroups are of approximately equal sizes), we find that the modular structure that leads to the lowest collective accuracy occurs very close to when there are $\sqrt{N}$ subgroups with $\sqrt{N}$ individuals per subgroup (figure 4*b*). Overall, then, the highest collective accuracy is achieved either with a large number of small subgroups *or* a small number of large subgroups (with a slight accuracy advantage to the former), although all groups with modular structure suffer a decrease in collective accuracy compared to groups with no modular structure.

## 5. Modular structure can improve accuracy in complex environments

In natural environments, animals typically do not make judgments completely independently of each other, as assumed in our analysis so far, and in models such as the Condorcet jury theorem. Instead, the opinions of animals are often correlated with each other to some degree, and in such scenarios, small group sizes have often been shown to maximize collective accuracy [16,18]. This is because increasing group size initially increases collective accuracy owing to the benefit of opinion aggregation (the ‘wisdom of crowds’), but at larger group sizes, the correlated cue increasingly dominates the collective decision, decreasing accuracy. Because our analysis so far has demonstrated that modular structure within groups can decrease the effective group size when making decisions (often by a substantial fraction), animals in groups may be able to exploit the information loss resulting from such structure in order to improve their collective accuracy in such complex environments (where the information perceived by individuals in the group may be correlated with each other).

To test this, we examined the impact of modular structure on collective decisions within environments containing two informational cues, one of which is sampled independently by individuals in the group (the ‘uncorrelated cue’) and one that provides identical information to all individuals in the group (the ‘correlated cue’; see §7 and refs. [16,17] for details of the model). In this model, the uncorrelated cue provides correct information with some probability *r*_*L*_ > 0.5, the correlated cue provides correct information with probability *r*_*H*_ ≥ 0.5 and individuals form an opinion by probabilistically selecting the uncorrelated cue with probability *p* and the correlated cue with probability 1 − *p*. This probabilistic use of cues is the ‘weighted average’ analogue when there are only two cues available, since an actual weighted average would trivially result in the more strongly weighted cue being used, and thus determining every decision.

**Figure 5.**
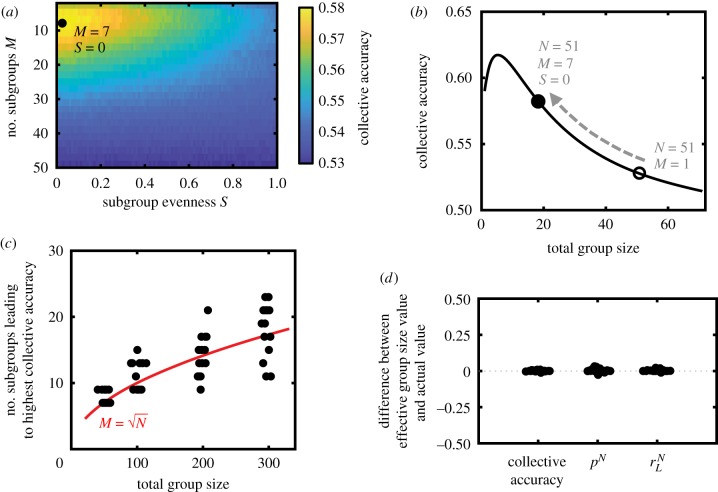
Modular structure can improve decision accuracy in complex environments. (*a*) In environments containing multiple informative cues, which vary in their degree of correlation across individuals in the group, a monotonic increase in collective accuracy as group size increases (as in the Condorcet jury theorem) is typically not observed; instead, a finite (and often small) group size maximizes accuracy. Large groups may exploit modularity to reduce their effective group size, thereby behaving like a smaller group and increasing collective accuracy. Shown is the collective accuracy achieved as function of the number of subgroups *M* and subgroup evenness *S*, for a group of size *N* = 51 and an environment where the correlated cue provides correct information with probability *r*_*H*_ = 0.5, the uncorrelated cue is correct with probability *r*_*L*_ = 0.65 and individuals follow the uncorrelated cue with probability *p* = 0.6 (see §7 for details of the model). A particular number of subgroups and subgroup evenness maximizes collective accuracy. (*b*) Using the optimal modular structure found in (*a*) allows the group of size *N* = 51 to behave like a group of size *N*_eff_ = 18.4, allowing the group to gain additional decision accuracy. (*c*) Assuming that subgroups in animal groups are approximately evenly sized (*S* = 1), the number of subgroups leading to the highest collective accuracy is approximately $M=\sqrt{N}$. For each group size, 20 random values of the reliability of the correlated cue *r*_*H*_, reliability of the uncorrelated cue *r*_*L*_, and probability that an individual follows the uncorrelated cue *p* were tested. (*d*) The effective group size measure not only accurately describes the collective accuracy of a group with modular structure, but also the probability that the decision is dominated by the uncorrelated cue (*p*^*N*^) and the probability that the group makes a correct decision when the decision is dominated by the uncorrelated cue ($r_{L}^{N}$). Fifty random sets of parameter values, with total group size selected from the range *N* ∈ [11, 101], number of subgroups *M* ∈ [3, *N* − 2], subgroup evenness *S* ∈ [0, 1], uncorrelated cue accuracy *r*_*L*_ ∈ [0.5, 0.7], correlated cue accuracy *r*_*H*_ ∈ [0.5, 0.7] and individual probability of using the uncorrelated cue *p* ∈ [0, 1/(2*r*_*L*_)]. (Online version in colour.)

We varied the number of subgroups and the evenness of the sizes of the subgroups, and simulated such groups making decisions for one particular group size and environment (*N* = 51, *r*_*L*_ = 0.65, *r*_*H*_ = 0.5, *p* = 0.6; for this model, a finite optimal group size exists if *p* < 1/(2*r*_*H*_); the parameters otherwise control the exact size of the optimal group and the collective accuracy of an infinitely large group). We find that there exists a unique number of subgroups and evenness that maximizes collective accuracy (figure 5*a*). For this scenario, forming seven subgroups with maximum unevenness (where all but one of the subgroups contain only one individual and the other subgroup contains the rest of the individuals) allows a group of size *N* = 51 to achieve an effective group size of *N*_eff_ = 18.4. Because collective accuracy in this environment is maximized with a group of size 5 (with no modular structure), the presence of modular structure allows a large group to behave much more similarly to an optimally sized group and consequently achieve a substantially higher collective accuracy (figure 5*b*).

Across group sizes and environmental conditions, we observed that maximizing subgroup unevenness typically improves accuracy in these scenarios. However, this extreme modular structure may not be realistically achievable for many animal groups in which the subgroups tend to be approximately evenly sized [46]. Therefore, to better understand how real animal groups may improve accuracy through modular structure, we subsequently fixed the subgroup evenness to its maximum value (such that subgroups are approximately evenly occupied) and located the number of subgroups that maximized accuracy across a wide range of environmental conditions. We find that the optimal number of subgroups increases with group size, and also closely follows the $M=\sqrt{N}$ line (figure 5*c*). Therefore, having $\sqrt{N}$ subgroups leads to the lowest collective accuracy when individuals have independent information (as in the Condorcet jury theorem), but often leads to the highest collective accuracy when individual opinions exhibit some degree of correlation.

While in the Condorcet jury theorem scenario the only relevant output is the collective accuracy of a subgroup or group, for the more complex scenario with multiple informative cues there are additional quantities of interest. One such quantity is the propensity of a group’s decision to be dominated by the uncorrelated cue. A group’s decision is dominated by the uncorrelated cue when the group decision is not dependent on whether the correlated cue provided correct or incorrect information. This occurs when either a majority of correct votes has already been reached by individuals using the uncorrelated cue (such that the collective decision is correct regardless of the correlated cue); or when there is a submajority of correct votes using the uncorrelated cue, but the size of the correlated cue voting bloc is too small to result in a majority of correct votes even if the correlated cue is correct (such that the collective decision is incorrect regardless of the correlated cue). In analogy to the individual probability of following the uncorrelated cue, we denote the probability that a group’s decision is dominated by the uncorrelated cue as *p*^*N*^.

A second quantity of interest is the collective accuracy of the group when the group’s decision is dominated by the uncorrelated cue. Because, as described above, there are two ways in which the uncorrelated cue can dominate a collective decision, how the probability of these two ways changes across environments or group size is not obvious. In analogy with the reliability of the uncorrelated cue for an individual, we denote the probability that a group’s decision is correct when it is dominated by the uncorrelated cue as $r_{L}^{N}$.

Thus far, we have mapped a group containing modular structure to an effective group size without modular structure using only the collective accuracy of the group. If a group with modular structure is truly equivalent to a (smaller) group without modular structure, then it should have not only the same collective accuracy, but also have the same probability of being dominated by the uncorrelated cue *p*^*N*^ and being correct when dominated by the uncorrelated cue $r_{L}^{N}$. We therefore examined, across a wide range of environmental scenarios and group sizes, the difference between the predicted values for all three quantities between a group with modular structure and one without modular structure at the effective group size. We find that the effective group size is indeed an accurate description of a modular group for all three quantities (figure 5*d*). Therefore, the effective group size is an accurate descriptor of how a group with modular structure behaves.

## 6. Discussion

Despite a considerable amount of empirical evidence that many social animal groups exhibit persistent modular structure, there exists very little theoretical understanding of its effect on a group’s decision-making abilities. Here, we showed that, by silencing the minority opinion within subgroups, modularity necessarily causes a loss of information. This information loss is subsequently reflected in the group decision and is well described by the effective group size measure that we introduced. In general, modular structure is detrimental to collective decision accuracy in simple environments, such as those described by the Condorcet jury theorem, but can often enhance decision accuracy in more complex, and naturalistic, environments that contain correlations in the informative cues sensed by animals.

Modular structure may therefore be a mechanism to allow large groups to behave much as a smaller group would when making a collective decision, but to simultaneously retain many other recognized benefits of living in a large group, such as predator deterrence [1,2,77]. For species that typically live in very large groups (and are therefore very far from the optimal group size for most environments), animals could minimize their effective group size by constructing approximately $M=\sqrt{N}$ subgroups for a group of size *N*. Experimental evidence has uncovered scaling relationships between the correlation length of individual movements and the spatial extent of a flock of starlings [49], suggesting that the behavioural rules that social animals follow may automatically scale the number of subgroups as the size of a group varies. Nevertheless, many animal groups exhibit a modular structure that is far from this potentially optimal structure, such as large groups of migrating ungulates consisting of many small subgroups of genetically related individuals. Because any modular structure will cause a substantial decrease in the effective group size, while tuning the precise number of subgroups has a more minor effect on collective accuracy (figure 4*a*), any group with modular structure will experience much of the benefits of such structure.

However, that $M=\sqrt{N}$ subgroups is typically optimal for environments containing correlated information, and least optimal for scenarios where individuals make independent judgments, is true only if the probability that an individual makes a correct decision is greater than 0.5. If this is not the case, then the opposite predictions will hold [78]. Thus, the specific predictions of the effect of internal structure on collective decision accuracy are sensitive to the specific informational environment in which the animal species in question makes decisions. For different species or contexts, we may predict a small, moderate or large number of subgroups to be optimal.

A common assumption in the collective decision-making literature is that more information leads to better decisions. Our examination of modular structure highlights that this may not be the case. Here, we have shown that silencing the opinions of some individuals, even randomly, may have a beneficial effect on decisions. When correlations between opinions exist, or when the information available to individuals is very poor (*r* < 0.5), then more information can be detrimental, and decreasing the amount of information used in a collective decision (and modular structure is one mechanism for achieving this) can improve the quality of decisions.

There are further potential complex interrelationships between the internal structure of a group, the structure of correlations in the information perceived by individuals in the group and the distributions of knowledge or expertise in the group. For example, individuals within a subgroup are typically physically proximate to each other and thus spatially localized, while the correlation of an informational cue also typically has some spatial correlation, and it is not clear how the spatial scales of the subgroups and the environmental information interact to affect collective accuracy [79]. Moreover, ‘uninformed’ individuals lacking relevant knowledge about the current decision may be distributed across the subgroups (e.g. juveniles in family groups [80]) or may comprise entire subgroups (e.g. fission–fusion groups [50] or mixed-species groups [81]). Whether and how collective decision-making may be affected by partitioning or distributing correlations or knowledge among subgroups is not yet understood but may reveal new strategies by which animals in groups may improve the accuracy of their collective decisions.

## 7. Methods

### (a) Generating a subgroup structure with a particular subgroup evenness

Our main model assumes *N* total individuals in the group, which are assigned to one of *M* subgroups. In order to create a structure with a certain subgroup evenness *S*, we calculate the smallest possible Shannon diversity *H*_min_ (where all but one of the subgroups have one member and the last subgroup contains the remaining individuals) and the largest possible Shannon diversity *H*_max_ (where the subgroups are of equal size, or as close as possible)—the desired Shannon diversity *H* is therefore defined as *H* = *S*(*H*_max_ − *H*_min_) + *H*_min_. A subgroup evenness of *S* = 0 implies that *H* = *H*_min_, while *S* = 1 implies *H* = *H*_max_. In order to generate a subgroup structure with the desired subgroup evenness *S*, we initially assign individuals to subgroups such that the subgroups are of equal size (or as close as possible) (i.e. we start with *S* = 1). We then randomly select a subgroup that contains more than two individuals, randomly select a second subgroup containing at least as many individuals as the first subgroup (if one exists) and move two individuals from the smaller to the larger subgroup (in order to maintain odd-sized subgroups), thus decreasing the subgroup evenness. We continue this process of semi-randomly moving individuals across subgroups until the desired Shannon diversity is achieved.

### (b) Making a consensus collective decision in the Condorcet scenario

Each individual casts a vote (which is correct with probability *r*) and each subgroup forms a consensus decision by combining the votes of the individuals assigned to that subgroup through simple majority rule. An overall group consensus is then formed by combining the decisions of the subgroups, also by simple majority rule.

### (c) Making a consensus collective decision in the multiple cues scenario

In this scenario, individuals have access to two sources of information; however, the two sources differ in the correlation of the information provided to each individual. For one source (the uncorrelated cue), each individual perceives independently sampled information from that source, which is correct with probability *r*_*L*_ (i.e. each individual flips a biased coin with probability *r*_*L*_, which determines whether that individual perceives correct or incorrect information from that source). For the other source (the correlated cue), all individuals in the group perceive the same information from this source, which is correct with probability *r*_*H*_ (i.e. the group flips a biased coin with probability *r*_*H*_ once, which determines whether all of the individuals in the group perceive correct or incorrect information from that source).

Individuals probabilistically select one of the two information sources to base their vote on, selecting the uncorrelated cue with probability *p* and the correlated cue with probability 1 − *p*. These individual votes are then combined within each subgroup, and the subgroup decisions combined into an overall group consensus in the same manner as in the Condorcet scenario.

### (d) Modelling a distribution of individual accuracies

In this model, we assume that individuals may have different probabilities of voting correctly. The model is identical to the Condorcet scenario, except that the individual accuracies may be different for each individual. We assume that individual accuracies are drawn from a normal distribution with a certain population mean and a standard deviation of 0.05.

### (e) Modelling weighted average decision-making among subgroups

In this model, we assume that larger subgroups are more influential to the overall collective decision compared to smaller subgroups. The model is identical to the Condorcet scenario, with one difference: instead of the subgroup decisions combined into an overall group consensus through simple majority rule (where each subgroup carries the same weight), the subgroup decisions are instead weighted by the size of each subgroup.

## Supplementary Material

The effect of group size, subgroup evenness, and individual accuracy on the effective group size, when individuals have non-identical accuracies

## Supplementary Material

The effect of group size, subgroup evenness, and individual accuracy on the effective group size, when subgroup decisions are weighted by the size of the subgroup.

## Supplementary Material

Matlab code to generate all figures

## Data accessibility

This article has no additional data.
