# Supplementary figures and images for: Modular structure within groups causes information loss but can improve decision accuracy
Source: Philos Trans R Soc Lond B Biol Sci. 2019 Apr 22;374(1774):20180378. doi: 10.1098/rstb.2018.0378 (PMC6553586; doi:10.1098/rstb.2018.0378)

A

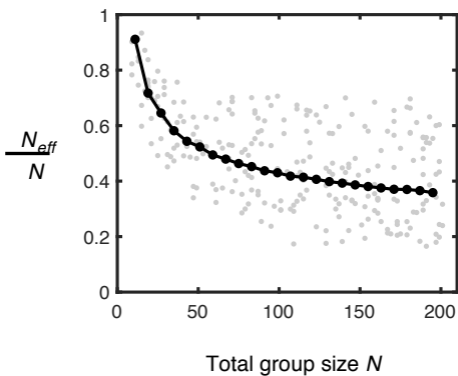

B

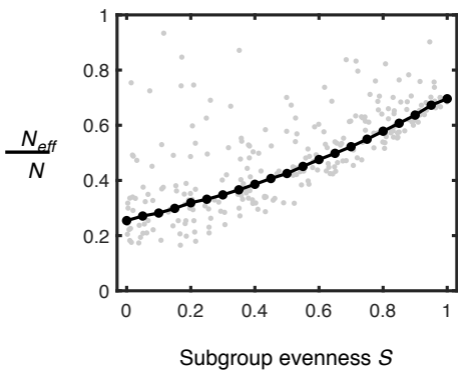

C

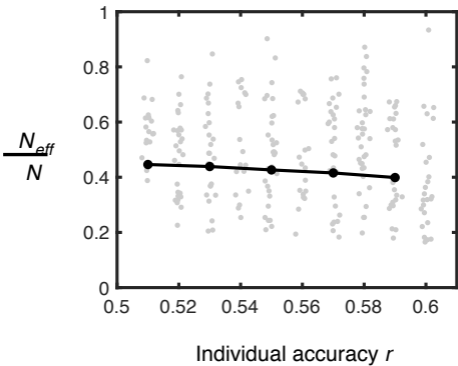

Supplement: The effect of group size, subgroup evenness, and individual accuracy on the effective group size, when individuals have non-identical accuracies [file rstb20180378supp1.pdf]

A

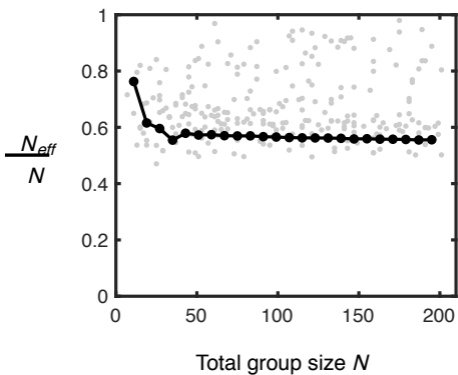

B

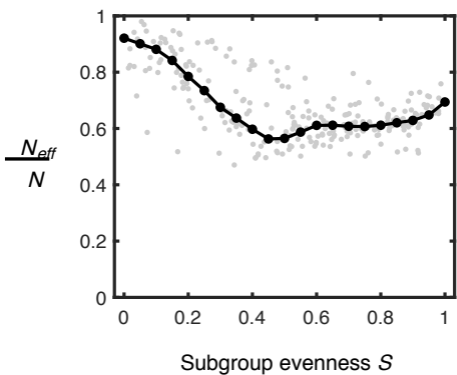

C

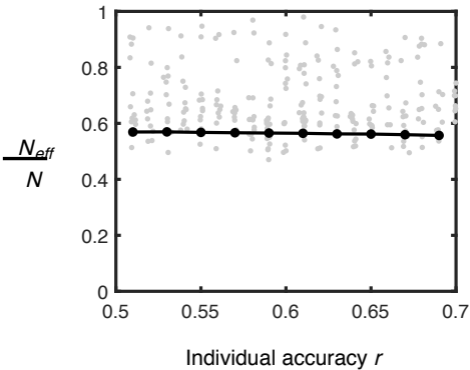

Supplement: The effect of group size, subgroup evenness, and individual accuracy on the effective group size, when subgroup decisions are weighted by the size of the subgroup. [file rstb20180378supp2.pdf]
